# Post-Traumatic Stress Disorder after Civilian Traumatic Brain Injury: A Systematic Review and Meta-Analysis of Prevalence Rates

**DOI:** 10.1089/neu.2018.5759

**Published:** 2019-11-11

**Authors:** Dominique L.G. Van Praag, Maryse C. Cnossen, Suzanne Polinder, Lindsay Wilson, Andrew I.R. Maas

**Affiliations:** ^1^ Department of Neurosurgery, Antwerp University Hospital and University of Antwerp, Edegem, Belgium.; ^2^ Center for Medical Decision Making, Department of Public Health, Erasmus MC, Rotterdam, The Netherlands.; ^3^ Division of Psychology, University of Stirling, Stirling, United Kingdom.

**Keywords:** civilian, epidemiology, post-traumatic stress disorder, systematic review, traumatic brain injury

## Abstract

Post-traumatic stress disorder (PTSD) is a commonly diagnosed psychiatric disorder following traumatic brain injury (TBI). Much research on PTSD and TBI has focused on military conflict settings. Less is known about PTSD in civilian TBI. We conducted a systematic review and meta-analysis on the prevalence of PTSD after mild and moderate/severe TBI in civilian populations. We further aimed to explore the influence of methodological quality and assessment methods.

A systematic literature search was performed on studies reporting on PTSD in civilian TBI, excluding studies on military populations. The risk of bias was assessed using the MORE (Methodological evaluation of Observational REsearch) checklist. Meta-analysis was conducted for overall prevalence rates for PTSD with sensitivity analyses for the severity of TBI.

Fifty-two studies were included, of which 31 were graded as low risk of bias. Prevalence rates of PTSD in low risk of bias studies varied widely (2.6–36%) with a pooled prevalence rate of 15.6%. Pooled prevalence rates of PTSD for mild TBI (13.5%, 95% confidence interval [CI]: 11.7–15.3; I^2^ = 2%) did not differ from moderate/severe TBI (11.8, 95% CI: 7.5–16.1; I^2^ = 63%). Similar rates were reported in studies using different approaches and times of assessment. Although most studies that compared participants with TBI with trauma patients and healthy controls found no difference in prevalence rates of PTSD, a meta-analysis across studies revealed a higher prevalence of PTSD in patients with TBI (odds ratio [OR]: 1.73, 95% CI: 1.21–2.47).

This review highlights variability between studies and emphasizes the need for higher-quality studies. Further research is warranted to determine risk factors for the development of PTSD after TBI.

## Introduction

Traumatic brain injury (TBI) is defined as an alteration in brain function, or other evidence of brain pathology, caused by an external force.^1^ Worldwide, up to 50 million people experience a TBI annually.^2^ In the European Union, the annual incidence of TBI is estimated at 2.5 million people, of whom 1.5 million are hospitalized and 57,000 die.^2,3^ Brain injury can have severe consequences on physical, cognitive, and affective functioning and may lead to long-lasting limitations in these domains.^4^

Psychological consequences for patients may require long-term rehabilitation, can result in difficulties in re-integrating into society, and can have a profound impact on families.^5^ Post-traumatic stress disorder (PTSD) is one of the most commonly diagnosed psychiatric disorders following TBI.^6–8^ According to the *Diagnostic and Statistical Manual of Mental Disorders*, fifth edition (DSM-5), PTSD is classified as a trauma-stressor-related disorder, rather than an anxiety disorder. After exposure to a traumatic event a diagnosis of PTSD based on DSM-5 requires symptoms from each of four clusters: intrusion, avoidance, negative alterations in cognitions and mood, and alterations in arousal and reactivity. Further, the symptoms must persist over 1 month, and in some cases people experience delayed-onset PTSD, which is defined as symptoms that appear 6 months or more after the traumatic event.^9^ The diagnosis requires significant distress and functional impairment in social or occupational setting. The disorder must be distinguished from disturbances due to medication, substance use, or other illness.^10^ *The International Classification of Diseases*, 10th edition (ICD-10) has a similar definition of PTSD.^11^

PTSD in a clinical setting is commonly diagnosed by a psychiatrist or psychologist after a (structured) interview. Structured interviews are usually seen as the gold standard but are rather time-consuming. For academic purposes both structured interviews and screening questionnaires are used. Questionnaires can be completed by the patient or by the clinician and measure symptoms determined by the DSM-5 or the ICD-10.^10,11^ For screening questionnaires a specific “triggering” event is not required, and PTSD cannot be unambiguously attributed to the events surrounding the TBI.

It is important to identify the occurrence of PTSD following TBI to provide timely and effective treatment. Early treatment can improve functioning and indirectly promote reintegration.^12^

Much research on PTSD and TBI has focused on military personnel in conflict settings.^13–15^ TBI among service members is often the result of a blast explosion or combat exposure in which emotional trauma is combined with physical trauma. The reported prevalence of PTSD in military personnel is high, ranging from 33 to 65%.^16,17^ In one study service members who lost consciousness during injury developed PTSD in 43.9% of cases, compared with 16.2% with other injuries or 9.3% without injuries.^13^ In a war setting individuals with blast-related TBI have higher numbers of PTSD symptoms than those with TBI due to other mechanisms.^18,19^

Less is known about the prevalence rates of PTSD in civilian TBI.^12,20,21^ Civilian TBI is typically caused by a motor vehicle accident, an assault, a sport injury, or a fall.^22^ Review articles show a wide range in estimates of prevalence of PTSD.^8,12,21,23–25^ Some studies report high rates of 24 to 50%,^26–28^ whereas others report low rates (3%)^29^ or no PTSD at all.^30,31^ An accurate estimation of prevalence is complicated by differences in assessment methods and methodologies.^12^ Previous reviews focused on mild TBI or made no differentiation of severity of TBI.

This systematic review was conducted to provide a comprehensive overview on PTSD following civilian TBI. The principal aim was to provide a valid estimate of the prevalence of PTSD following TBI in civilian populations, and to explore possible differences in prevalence between mild versus moderate/severe TBI. Secondary objectives were to assess the influence of assessment methods (e.g., (semi)-structured interviews versus questionnaire assessments) and to evaluate the methodological quality of studies, aiming to provide recommendations for the way forward in this research field.

## Methods

### Data sources and search strategy

A review protocol was published on PROSPERO, an online repository of systematic review protocols (ID 42016029956, available from: http://www.crd.york.ac.uk/PROSPERO/display_record.php?ID=CRD42016029956). The following databases were examined using a combination of MeSH/EMTREE terms and text words: MEDLINE, PsychINFO, EMBASE, CINAHL, Cochrane, and Web of Science (Supplementary Appendix S1). We searched for gray literature using Web of Science Conference Proceedings, Google Scholar, opengrey.eu, Behavioral and Brain sciences – Cambridge University Press, BioMed Central, and the National Institute of Mental Health. Further, we searched the reference lists of the included studies for additional relevant studies.

### Selection criteria

We included prospective and retrospective observational studies, such as cohort studies, case-control studies, and cross-sectional studies. All studies that examined patients with TBI in a civilian population were included. Studies with mixed populations, for example TBI and non-head injury or military TBI and civilian TBI, were included if the authors analyzed the results of civilian TBI separately. Studies that focused only on military personnel were excluded. All studies that measured mental health symptoms of PTSD were included, irrespective of how the PTSD was diagnosed and at what time-point after the TBI. We included studies that used self-report questionnaires or (semi-)structured interviews. Only articles published after 1980 were included because the diagnosis of PTSD was described in 1980 in the *Diagnostic Manual of Mental Disorders* (DSM-III) for the first time.^32^ We included articles written in English, French, or Dutch.

### Data extraction

Two authors independently screened all titles and abstracts identified in the searches and determined whether they were eligible for inclusion. Relevant articles were retrieved in full text and screened independently by the same two authors for the final review and data extraction. Disagreement was resolved by discussion until consensus was reached or by consulting a third author. The Covidence platform was used for the screening and selection of studies for the review.^33^ Articles with the same sample of patients were identified to avoid double counting of prevalence rates, and only the study with the largest sample size was included.

The following data were extracted: study characteristics (study design, year of publication), setting (country), participants (number, age, severity of TBI, control population), and outcome (type of PTSD instrument, time-point after TBI, percentage with PTSD).

### Quality assessment

The methodological quality of the studies was evaluated with the checklist for Methodological evaluation of Observational REsearch (MORE).^34^ This checklist contains six criteria for external validity and five criteria for internal validity. External validity is the extent to which results can be generalized to the population and contains the evaluation of sampling strategies, sampling bias, estimate bias, exclusion rate from the analysis, and address bias. The internal validity is the extent to which the results are correct for the subjects in the study and contains the evaluation of source of measure, definition of measure, validation of measures and reliability of the estimates, definition of outcomes in subpopulations, and reporting of prevalence. Each domain was rated as Good, Minor flaw, Major flaw, or Poorly reported according to the MORE criteria.^34^ Studies that had one major flaw were classified as “Moderate risk of bias.” Studies that had more than one major flaw, or one major flaw plus at least two minor flaws were classified as “High risk of bias.” Studies without major flaws but with minor flaws in three or more domains were classified as “Moderate risk of bias” as well. All other studies were classified as “Low risk of bias.” Two authors used the checklist to rate each study independently. Any disagreement was resolved by discussion until consensus was reached or by consulting a third author.

### Statistical analysis

The studies were grouped by severity of TBI (mild, moderate, severe, and mixed severity) as defined in the reports. Criteria used by studies varied, but were mostly based on the Glasgow Coma Scale (GCS: mild 13–15, moderate 9–12, severe 3–8), post-traumatic amnesia (PTA: mild TBI <24 h, moderate and severe >24 h) and loss of consciousness (LOC: mild <20 or 30 min). Studies were grouped by type of measurement (self-report questionnaire, semi-structured interview, or structured interview) and time-point of measurement (3, 6, 12, and over 12 months up to 5 years post-injury). The Comprehensive Meta-Analysis Software was used to perform a meta-analysis including a sensitivity analysis using the chi-square test and computed the I^2^-statistic. An I^2^-value of 50 or more was considered to represent substantial levels of heterogeneity.

## Results

### Literature search

The systematic search identified 7,607 articles. After removing duplicates, titles and abstracts of 4381 unique articles were screened. Full-text screening of 744 relevant articles yielded 52 eligible studies, described in 55 publications (Supplementary Fig. S1).

## Study characteristics

Approximately one-third of all included studies were conducted in each of the following geographic areas: Australia (*n* = 16),^7,35–49^ the United States (*n* = 16),^50–65^ and Europe (*n* = 15)^66–80^ (Supplementary Table S1). Almost half of the studies had a small sample size (<100; *n* = 25) and only seven studies had a large sample size (500–3000). The percentage of male participants ranged from 20.5 to 100%; nearly half of the studies included more than 70% males (*n* = 23). The mean age of the participants ranged from 24 to 50 years of age with an average of 33 to 37 in almost half of the studies (*n* = 24). Nearly half of the studies did not define the severity of injury (*n* = 24). Five studies described including two or three levels of severity and reported outcome for each subgroup.^50,54,61,62,65^ The remaining studies were focused on patients with mild TBI (*n* = 13),^7,38,41,42,47,48,59,66,70,72,74,76,81^ moderate and severe TBI (*n* = 4),^35,36,46,52^ and severe TBI (*n* = 6).^40,67,77,78,80,82^ Most studies were prospective cohort studies (*n* = 33) that recruited patients at the time of TBI with follow-up for PTSD at a later time-point. Fourteen studies measured PTSD at one time-point in patients with a history of TBI, using a cross-sectional study design. Three studies used a retrospective cohort design^49,54,59^ and two a case-control design.^55,75^

A total of 77 different outcome measurements were reported in the 52 studies at different time-points, ranging from the time of injury up to 30 years after TBI. Forty-nine of the outcome assessments reported were performed within the first year after TBI, of which 32 were within the first 6 months.

## Methodological quality

Of the 52 studies, a total of 31 studies were graded as having a low risk of bias. Six studies had major flaws for external validity, 11 for internal validity, and four for both external and internal validity, resulting in 21 unique studies with a moderate or high risk of bias (Supplementary Table S2). The external validity showed the following methodological problems: six studies faced problems with estimating bias (response rates 14%–33%),^44,49,54,71,79,83^ three studies with a high exclusion rate from the analysis (43–71%),^35,48,55^ and three studies with subject sampling.^54,63,83^ The assessment of sampling bias and address bias were poorly reported in 14 and 19 studies, respectively. Twenty studies did not report response rates. Most of the studies with low internal validity had no or an unclear definition of PTSD (*n* = 11) or used unvalidated measures (*n* = 4). None of the studies showed problems with the source of measure or reporting the prevalence of PTSD. In 40 and 47 studies, respectively, the reliability of the estimate and the precision of estimate were poorly reported.

## Prevalence rates and comparison with non-TBI trauma populations and healthy controls.

**FIG. 1.**
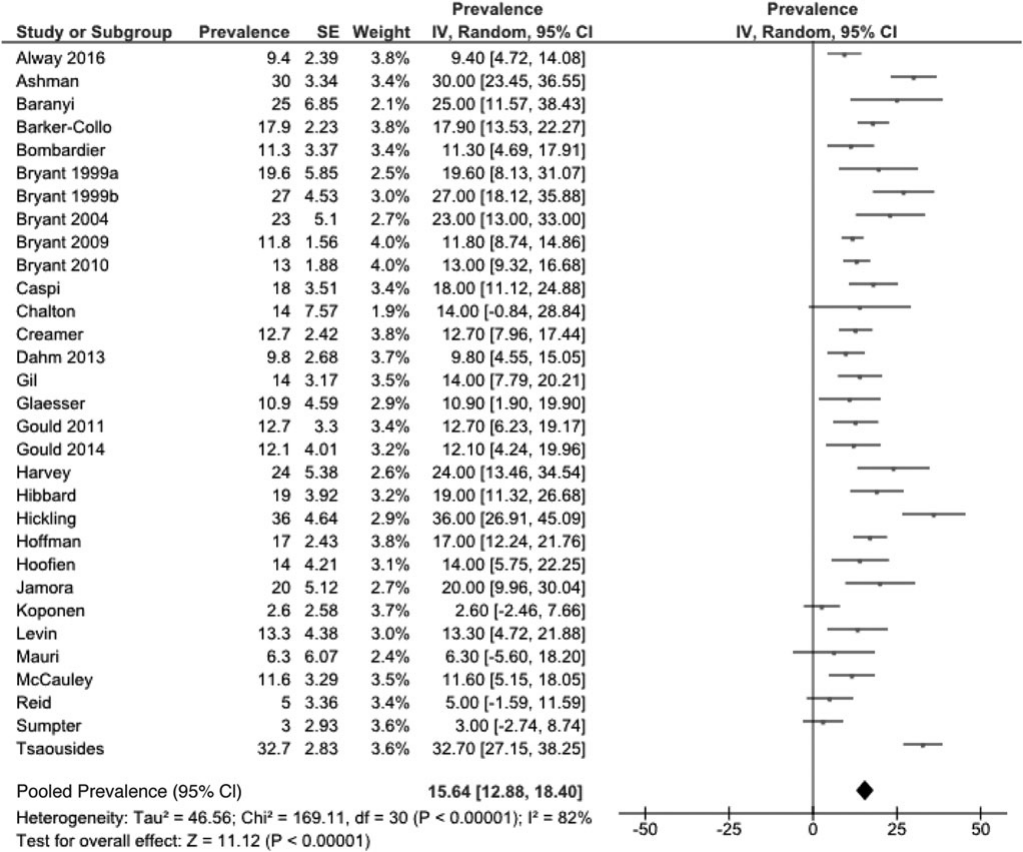
Meta-analysis of PTSD in TBI in all low risk of bias studies. PTSD, post-traumatic stress disorder; TBI, traumatic brain injury.

Prevalence rates of all 52 studies range from 0 to 36%, and remain wide for the 31 studies with a low risk of bias (2.6–36%). A meta-analysis of the latter gave a pooled prevalence rate of 15.6% (95% CI: 12.8–18.4); however, the heterogeneity was high (I^2^ = 82, *p* < 0.01) (Fig. 1).

**FIG. 2.**
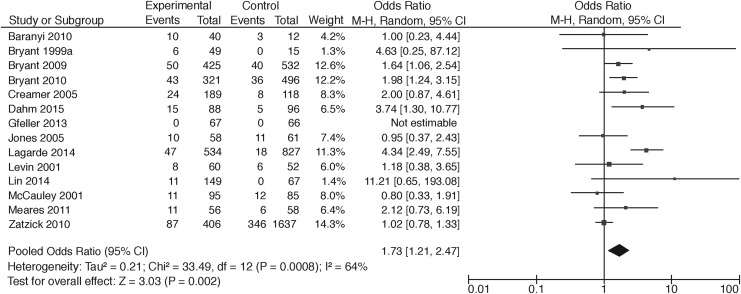
Meta-analysis of PTSD rates comparing TBI patients with non-brain trauma patients. PTSD, post-traumatic stress disorder; TBI, traumatic brain injury.

**FIG. 3.**
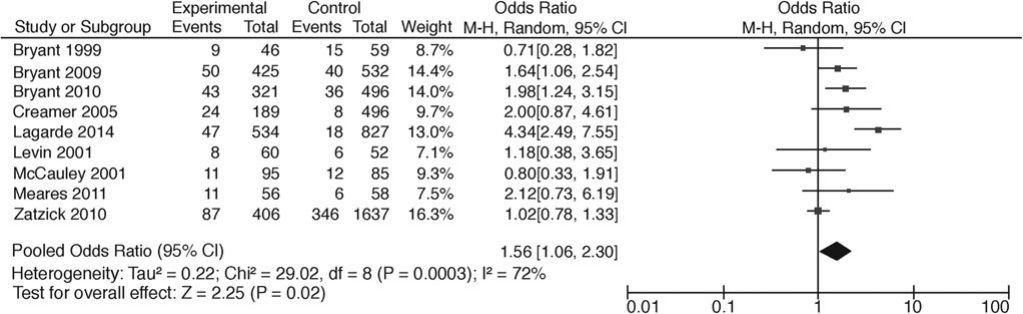
Meta-analysis of PTSD rates comparing mild TBI patients with non-brain trauma patients. PTSD, post-traumatic stress disorder; TBI, traumatic brain injury.

Thirteen studies compared participants with TBI with trauma patients without TBI and one study used a matched healthy control group. Of these, Zatzick and colleagues^65^ reported PTSD rates for mild, moderate, and severe TBI separately, Levin and associates^61^ and McCauley and co-workers^62^ reported prevalence rates of PTSD for mild and moderate TBI, resulting in 18 different comparisons. Two studies did not report whether the difference between prevalence rates was significant. In most studies, the difference between the group with TBI and the group without TBI was not statistically significant (*n* = 10), of which one study showed a floor effect.^55^ Six studies did report a significant difference, with higher percentages for mild TBI compared with no TBI (*n* = 3), lower percentages for moderate or severe TBI compared with no TBI (*n* = 2), and a higher percentage for TBI (severity undefined) compared with a group with traumatic orthopedic injury (Table 1). A meta-analysis of all studies comparing TBI patients with non-brain injury patients showed a pooled odds ratio (OR) of 1.73 (95% CI: 1.21–2.47) (Fig. 2). A meta-analysis of all studies comparing mild TBI with a trauma patient group without TBI showed a pooled OR of 1.56 (95%CI: 1.06–2.30) (Fig. 3). Interpretation should, however, be made with caution given the large heterogeneity (respectively, I^2^ = 64, *p* < 0.01 and I^2^ = 72, *p* = 0.02).

**Table 1.** Percentage of PTSD in Studies with a Control Group

*Study, year*	*Time of assessment*	*TBI patients*	N *PTSD/*N *total (%)*	*Control group*	N *PTSD/*N *total (%)*	*OR (95% CI) and/or* p*-value*
Bryant, 1999^26^	6 months	Mild TBI	9/46 (19.6)	Traumatic injury no TBI: motor vehicle accidents	15/59 (25.4)	NR
Bryant, 2009^41^	3 months	Mild TBI	50/425 (11.8)	Traumatic injury no TBI	40/532 (7.5)	NR
						OR 1.86 (CI = 1.78–2.94)
Bryant, 2010^7^	12 months	Mild TBI	43/321 (13.4)	Traumatic injury no TBI	36/496 (7.3)	0.004
Creamer, 2005^42^	12 months	Mild TBI	24/189 (12.7)	Traumatic injury no TBI	8/118 (6.8)	0.100
Lagarde, 2014^74^	3 months	Mild TBI	47/534 (8.8)	Mild traumatic injury no TBI: sprains, contusions, or fractures; no intrathoracic or intra-abdominal injuries	18/827 (2.2)	NR
						OR 4.47 (CI = 2.38–8.40)
Levin, 2001^61^	3 months	Mild TBI	8/60 (13.3)	Traumatic injury no TBI	6/52 (11.5)	NS
McCauley, 2001^62^	3 months	Mild TBI	11/95 (11.6)	Traumatic injury no TBI	12/85 (14.1)	NS
Meares, 2011^48^	3 months	Mild TBI	11/56 (19.6)	Traumatic injury no TBI	6/58 (10.3)	NS
Zatzick, 2010^65^	12 months	Mild TBI	87/406 (21.4)	Traumatic injury no TBI	346/1637 (21.1)	NR
						RR 0.83 (CI = 0.61–1.13)
Levin, 2001^61^	3 months	Moderate TBI	0/9 (0.0)	Traumatic injury no TBI	6/52 (11.5)	NR
McCauley, 2001^62^	3 months	Moderate TBI	4/20 (20.0)	Traumatic injury no TBI	12/85 (14.1)	NS
Zatzick, 2010^65^	12 months	Moderate TBI	67/358 (18.7)	Traumatic injury no TBI	346/1637 (21.1)	NR
						RR 0.63 (CI = 0.44–0.89)
Baranyi, 2010^67^	12 months	Severe TBI	10/40 (25.0)	Traumatic injury no TBI; ISS >11	3/12 (25.0)	NS
Zatzick, 2010^65^	12 months	Severe TBI	102/592 (17.2)	Traumatic injury no TBI	346/1637 (21.1)	NR
						RR 0.72 (CI = 0.58–0.90)
Dahm, 2015^44^	5 to 10 years	TBI-SU	15/88 (17.0)	Traumatic orthopedic injury	5/96 (5.2)	0.002
Gfeller, 2013^55^	Mean 51 months	TBI-SU	0/67 (0.0)	Healthy civilian control group	0/66 (0.0)	NS
Jones, 2005^71^	3 months	TBI-SU	10/58 (17.2)	Traumatic injury no TBI	11/61 (18.0)	NS
Lin, 2014^83^	12 months	TBI-SU	11/149 (7.4)	Crushing injuries; open wound of upper limbs; fractures; burns	0/67 (0.0)	NS
					1/84 (1.6)	
					18/698 (2.6)	
					1/27 (3.7)	

*P* > 0.05; OR and RR as reported by authors.

CI, confidence interval; ISS, Injury Severity Score; MVA, motor vehicle accident; NR, not reported; NS, not significant; OR, odds ratio; RR, relative risk; TBI-SU, traumatic brain injury severity undefined.

### Subanalysis by TBI severity

**FIG. 4.**
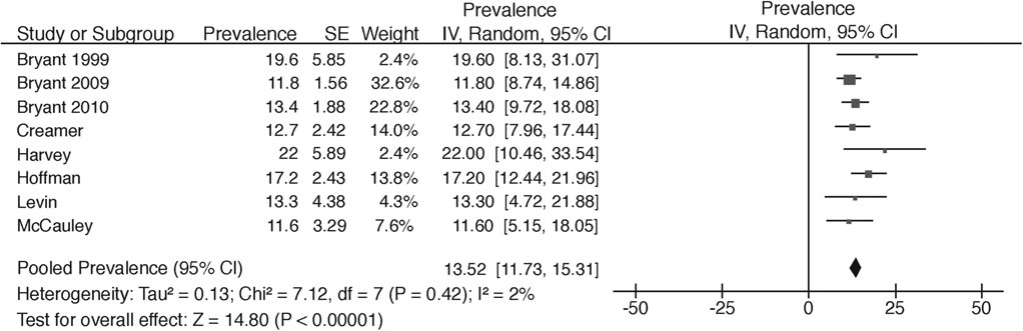
Meta-analysis of PTSD in mild TBI in low risk of bias studies. PTSD, post-traumatic stress disorder; TBI, traumatic brain injury.

Eighteen studies included patients with mild TBI and reported 23 prevalence rates across different time-points. Prevalence rates ranged from 4 to 34%. Four studies found rates up to 10%, nine studies reported percentages from 11 to 20%, three studies showed prevalence rates from 21 to 30%, and two studies reported a percentage higher than 30%. Almost half of the studies were graded as having a low risk of bias (*n* = 8) and meta-analysis showed a pooled prevalence rate of 13.5% (I^2^ = 2%, *p* = 0.42) (Fig. 4). This was not substantially different from the meta-analysis of all studies (14.8%, 95% CI: 12.0–17.5; I^2^ = 79%, *p* < 0.01) (Supplementary Fig. S2.1), but confidence intervals were narrower (11.6% to 22%) compared with those for all studies.

**FIG. 5.**
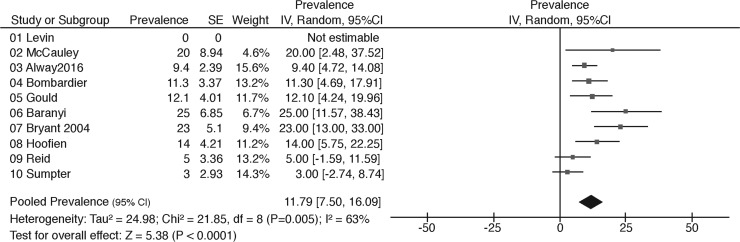
Meta-analysis of PTSD in moderate and severe TBI in low risk of bias studies. PTSD, post-traumatic stress disorder; TBI, traumatic brain injury.

Sixteen studies reported on the prevalence of PTSD in patients with moderate and severe TBI. Three studies included exclusively moderate TBI, five studies included a mixed population of moderate and severe TBI, and eight studies reported on severe TBI only. Combined, the studies reported 27 different prevalence rates at different time-points. Prevalence rates ranged from 0 to 25%. Ten studies had a low risk of bias. Meta-analysis of these studies showed a pooled prevalence rate of 11.79% (I^2^ = 63, *p* < 0.01; Fig. 5), which does not differ substantially from the finding from meta-analysis of all studies (13.41%, 95% CI = 10.0–16.8; I^2^ = 74, *p* < 0.01) (Supplementary Fig. S2.2).

Twenty-four studies with a mixed severity group reported a total of 29 prevalence rates at different time-points. The reported percentage of PTSD following TBI varied from 0 to 36%. Six studies reported percentages up to 10%, 13 studies showed prevalence rates from 11 to 20%, two studies showed rates from 21 to 30%, and three studies found rates above 30%. Fifteen studies were graded as having a low risk of bias. Meta-analysis of these studies showed an overall prevalence rate of 17.8% (95% CI = 12.5–23.0) (Supplementary Fig. S2.3). This was not substantially different from meta-analysis of all studies (16.6%, 95% CI = 11.5–21.6) (Supplementary Fig. S2.4). Heterogeneity was high for both studies having a low risk of bias (I^2^ = 88%, *p* < 0.01) and all studies (I^2^ = 95%, *p* < 0.01).

### Method of assessment

Different types of instruments were used to report on symptoms of PTSD (Table 2). Half of the studies used a (semi-)structured interview (*n* = 27), 17 studies used a questionnaire, and eight studies used a questionnaire and a (semi-)structured interview. Three studies compared the results of the questionnaire and the interview and found a lower prevalence of PTSD when using (semi-)structured interviews compared with self-report questionnaires.^68,78,79^

**Table 2.** Assessments for PTSD

*Self-report questionnaire*	N	*Prevalence rates/ranges, %*	*Semi-structured interview*	N	*Prevalence rates/ranges, %*	*Structured interview*	N	*Prevalence rates/ranges, %*
PCL: PTSD Checklist	9	0–21.4	SCID: Structured Clinical Interview for the DSM	15	0–32.7	CAPS: Clinician-Administered PTSD Scale	12	3–36
IES: Impact of Event Scale	9	3–34	PSS: Post-traumatic Symptom Scale	2	14–17.2	PTSD-I: Post-traumatic Stress Disorder Interview	3	14–33.3
PDS: Post-traumatic Diagnostic Scale	3	3–33	SCAN: Schedules for Clinical Assessment in Neuropsychiatry	1	2.6	CIDI: Composite International Diagnostic Interview	2	19.6–24
RNBI: Ruff Neurobehavioral Inventory	1	20	not specified	3	3–27.5	MINI: Mini International Neuropsychiatric Interview	1	7.5
HTQ: Harvard Trauma Questionnaire	1	31						
PTSC: Post-traumatic Symptom Checklist	1	7.5						
not specified	1	8.8						

Six different instruments were used for questionnaire assessments, of which the PTSD Checklist (PCL)^84,85^ and the Impact of Events Scale (IES)^86^ were the two most frequently used (Table 2). Nine studies used the PCL to measure PTSD following TBI and showed rates from 0 to 21.4% of symptoms of PTSD. Almost all studies used the PCL-C (civilian version) based on the DSM-IV,^87^ and only one study used the PCL-5, based on the DSM-5 published in 2013.^85^ Three studies used the symptom cluster method (SCM)^88^ by executing an algorithm for the symptom items to meet the criteria of the DSM-IV.^89^

In 11.3 to 17.2% of the cases PTSD was diagnosed. Studies that used a cutoff score to differentiate between high risk of PTSD and low risk of PTSD showed high PTSD symptoms in 0 to 21.4% of the patients. One study did not report how the PCL was scored or interpreted.^53^ Levin and colleagues^61^ and McCauley and associates^62^ used the Structured Clinical Interview for the DSM I (SCID-I) to diagnose PTSD and assessed the severity of PTSD using the PCL (Table 3).^90,91^ Nine studies used the IES, of which five used this as the only measure to assess PTSD and showed prevalence rates from 8.5 to 34%.^86^ The quality of the studies was rated as moderate because they used a screening instrument that does not qualify to diagnose PTSD. Besides this criterion, the studies did not show other flaws, and therefore the quality assessment had no impact on the range of prevalence rates. Studies that used a lower cutoff score (≥26) showed higher prevalence rates (9.5 to 34%) compared with studies that utilized a cutoff of 35 and higher (8.5 to 16.2%) (Table 3).

**Table 3.** Prevalence Rates Measured by PCL, IES, CAPS, and SCID

*Author, year*	*Scoring*	*PTSD prevalence rate* n/N *(%)*	
*Screening questionnaire: PTSD Checklist*			
Bombardier, 2006^52^	SCM		14/124 (11.3)
Choi, 2014^81^	Cutoff score = 44		10/71 (14.1)
Dahm, 2015^44^	SCM		15/88 (17.0)
*Dams-O'Connor, 2013^53^*	*NR*		*4/586 (0.7)*
Gfeller, 2013^55^	Cutoff score (score NR)		0/67 (0.0)
*Hoffman, 2012^59^*	*SCM*		*41/239 (17.2)*
*Levin, 2001^61^*	*Total score to assess severity*	*mTBI*	*8/60 (13.3)*
		*modTBI*	*0/9 (0.0)*
*McCauley, 2001^62^*	*Total score to assess severity*	*mTBI*	*11/95 (11.6)*
		*modTBI*	*4/20 (20.0)*
*Zatzick, 2010^65^*	*Cutoff score ≥45*	*mTBI*	*87/406 (21.4)*
		*modTBI*	*67/358 (18.7)*
		*sTBI*	*102/592 (17.2)*
*Screening questionnaire: Impact of Event Scale*			
*Ahman, 2013^66^*	*Cutoff score ≥26*		*Men 9/95 (9.5)*
			*Women 9/68 (13.2)*
Greenspan, 2006^56^^*^	*Cutoff score ≥35*		*32/198 (16.2)*
Haagsma, 2015^70^^*^	*Cutoff score ≥35*		*24/282 (8.5)*
*Powell, 1996^76^*	*Cutoff score >26*		*12/35 (34.0)*
*Williams, 2002^80^*	*Cutoff score ≥26*		*12/66 (18.2)*
*Semi-structured interview: Structured Clinical Interview for the DSM*			
Alway, 2015^35^^*^			*6/85 (7.0)*
Alway, 2016^36^^*^			*14/149 (9.4)*
Ashman, 2004^51^^*^			*56/188 (30)*
*Dahm, 2013^43^*			*12/123 (9.8)*
*Glaesser, 2004^69^*			*5/46 (10.9)*
*Gould, 2011^45^*			*13/102 (12.7)*
*Gould, 2014^46^*			*8/66 (12.1)*
*Hibbard, 1998^57^*			*19/100 (19.0)*
Mauri, 2014^75^^*^			*0/16 (0.0)*
*Tsaousides, 2011^64^*			*90/275 (32.7)*
*Whelan-Goodinson, 2009^49^*			*14/100 (14.0)*
*Structured interview: Clinician-administered PTSD Scale*			
*Bryant, 2009^41^*			*50/425 (11.8)*
Bryant, 2010^7^^*^			*43/321 (13.4)*
*Chalton, 2009^68^*			*3/21 (14.0)*
*Creamer, 2005^42^*			*24/189 (12.7)*
*Hickling, 1996^58^*			*38/107 (36.0)*
*Meares, 2011^48^*			*11/56 (19.6)*
*Reid, 2011^77^*			*2/42 (5.0)*

^*^ In studies with multiple time-points, the table shows measurement at 12 months.

Studies in italics are low risk of bias.

CAPS, Clinician-Administered PTSD Scale; DSM, *Diagnostic and Statistical Manual of Mental Disorders*; IES, Impact of Event Scale; NR, not reported; modTBI, moderate traumatic brain injury; mTBI, mild traumatic brain injury; sTBI, severe traumatic brain injury; PCL, PTSD Checklist; PTSD, post-traumatic stress disorder; SCID, Structured Clinical Interview for the DSM; SCM, symptom cluster method.

Three different semi-structured interviews and three structured interviews were used. The SCID was used as a measure for PTSD in 11 studies.^90,91^ The prevalence rates varied from 0 to 32.7%, even when excluding studies with a moderate risk of bias. Seven studies used the Clinician-Administered PTSD Scale (CAPS) to diagnose PTSD and reported eight prevalence rates from 5 to 36%.^92,93^ The only study including exclusively severe TBI presented a prevalence of 5% PTSD, whereas the study reporting a prevalence of 36% only included motor vehicle accidents.^58^ Excluding the study with a moderate risk of bias did not change the prevalence interval (Table 3).

### Time-point of measurement

The reported prevalence of PTSD measured at 3 and 6 months post-injury showed rates from 1.9 to 36.0%. and 0.0 to 33.3%, respectively. Six studies reported a percentage below 10%, 12 studies showed rates from 11 to 20%, four studies showed a percentage between 21 and 30%, and two studies measured a percentage above 30%. Measurements at 12 months post-injury showed rates varying from 2.6 to 21.4%. This does not decrease when adding measurements up to 5 years post-injury (2.4–22%). In five studies, the authors found rates up to 10%, 12 studies showed a rate of 11 to 20%, and one study reported a percentage of 21 to 30%.

Evidence suggests that prevalence remains relatively high even many years after injury. Nine studies performed follow-up assessments of PTSD up to 5 years post-injury. However, no discernible patterns were found in patients with TBI who were followed longitudinally. Over the first 6 months two studies showed worsening of symptoms of PTSD,^35,36^ two studies showed an improvement of the symptoms,^51,75^ and a larger study by Bryant and colleagues showed no change over time.^7^ For the next 6 months after injury (6 to 12 months) no clear increase or decrease of symptoms can be deducted. Four studies showed an increase in symptoms over time,^35,36,54,56^ three studies showed a decrease in symptoms,^47,51,54^ and two larger studies showed no change in symptoms.^7,70^ Ashman and associates reported a worsening after 12 months up to 2 years post-injury,^51^ but other studies showed improvement of symptoms of PTSD over time.^35,36,47^ The findings suggest that multiple factors need to be taken into account when drawing conclusions concerning prevalence rates and change over time.

## Discussion

The topic of PTSD after TBI has attracted much public attention in the past decade and has been extensively studied in military populations. Prevalence rates of PTSD of up to 65% have been reported in military populations in which repeated injuries are not uncommon and exposure to stressful events is likely.^94^ Less is known about PTSD in the civilian population, where injury mechanisms and circumstances are vastly different. To our knowledge, this is the first systematic review on the occurrence of PTSD following mild, moderate, and severe TBI in civilians. Prevalence rates of PTSD ranged from 0 to 36% over the 52 different studies with a pooled prevalence rate of 15.6% in studies with a low risk of bias. Pooled prevalence rates were not markedly different for mild TBI (13.5%) compared with moderate and severe TBI (11.8%). Studies using screening questionnaires showed similar percentages of PTSD compared with those that used diagnostic interviews, a finding which is not consistent with previous research.^68,78,79^ However, studies that utilized both questionnaire and interview assessment reported higher prevalence rates with the use of a questionnaire. Reported prevalence rates of PTSD were similar at 3, 6, and 12 months, even up to 5 years after TBI, but the intervals of prevalence rates were wide and studies were not comparable.

### PTSD after TBI

Several studies compared PTSD rates between patients with a TBI with non-brain injury trauma patients and most studies found no significant difference in prevalence rates. Meta-analysis across these studies did reveal a higher prevalence of PTSD in patients with TBI. This lends some support to the concept that TBI-specific factors, such as disruption in brain circuitry, may underlie the comorbidity of TBI and PTSD.^95^ The high heterogeneity between studies, however, prevents drawing strong conclusions. We found no clear effect of severity on prevalence rates, with 13.5% and 11.8% for patients with a mild TBI and patients with a moderate or severe TBI, respectively. This would suggest that besides TBI-specific factors, such as disruption of brain circuitry, non-TBI specific factors (e.g., pre-trauma factors, patient- or event-related factors) might play a role in the development of PTSD after TBI.^96^

The stressful event itself is clearly of importance in developing PTSD, and relates not only to the traumatic incident, but also, for example, to the loss of a loved one or colleague in the event or to the treatment following the injury.^97^ A few studies recorded the cause of injury but found no significant difference in PTSD for indoor falls, outdoor falls, falls from a height or a bicycle, horseback riding, assaults, or vehicle related or sports related injuries.^37,52,59,67,81^ Nonetheless, three studies only included motor vehicle accidents and reported high prevalence rates ranging from 19.6 to 36%.^47,54,58^ Reported prevalence rates of PTSD following TBI are higher in military populations, possibly reflecting repeated trauma and exceptionally traumatic circumstances compared with civilian populations.^16,17^ Future research should focus on pre-trauma-, event-, and patient-related factors, their interaction with biological factors and determination of risk factors for the development of PTSD.^97^

### Interaction between the consequences of TBI and PTSD

Although the brain injury itself seems to play at most only a minor role in the development of PTSD, the combination of PTSD and TBI with its consequences will cause more difficulties in diagnosing and treating PTSD. TBI and PTSD have many overlapping symptoms, including sleep disturbance, irritability, memory and concentration difficulties, fatigue, nausea, depression, headaches and reduced speed of information processing.^98^ Interactions may occur between symptoms of TBI and PTSD: loss of memory for the event itself might be protective, whereas cognitive problems caused by TBI can have negative consequences for coping capacities, create more anxiety and stress, and therefore cause a higher risk of developing PTSD. Conversely, patients developing PTSD often have prominent cognitive complaints and this may lead to an over-estimation of PTSD.^98^ Twenty studies took this into account and performed neuropsychological assessment in their patient population, of which eight studies solely performed a self-report measurement of cognitive complaints. Cognitive difficulties are a major concern for researchers studying psychological outcome because questions might be answered impulsively and attention deficits may induce inconsistent responses.^98,99^

### Time since injury

PTSD can only be formally diagnosed when symptoms last for more than one month. Most studies research PTSD within the first 6 months after the injury, whereas the prevalence of PTSD remains high after a longer period of time. The overall results show no clear decrease in the prevalence of PTSD; however, high variability between studies and presumably loss to follow-up prevent reliable conclusions. This finding contrasts with early studies that showed a time-dependent decline in the frequency of PTSD symptoms.^100–102^ Only two studies in this review showed prevalence rates declining from 30 to 21% over 3 years, and 18 to 14% over 15 years.^51,66^ Our results support the findings of Carlson and colleagues that show no specific patterns over time in longitudinal studies.^12^ Symptom trajectories following exposure include both recovery and worsening, which could explain the unaltered interval of prevalence rates. The challenge will be to identify the risk to patients for the development of PTSD and to differentiate between the recovery-trajectory patients and the worsening-trajectory patients.^103^

### Limitations

This review is limited by the deficiencies in the underlying studies, for example, missing or unreported values. To estimate the quality of the studies, more information is necessary on how samples were included, response rate and the reason for low response rate, the definition of TBI and PTSD, the psychometric properties of the instruments, and the reliability of outcome. Studies using a questionnaire are limited in attributing a diagnosis of PTSD because they do not require the symptoms to be related to a specific traumatic event, nor do they require the symptoms to last at least 1 month. Additionally, half of the studies did not identify levels of severity, which means the study samples are quite heterogeneous. The results of self-report questionnaires may sometimes be unreliable for TBI patients because of concentration deficits caused by TBI, impulsivity, or the tendency of underestimating their functional problems.^21^ Special attention or help to answer the questionnaire may be expected to yield more accurate results. For each new edition of the DSM, every questionnaire or interview is reviewed and a new version is issued.^10,104^ Earlier studies using a measurement related to the DSM-III show higher prevalence rates, compared with instruments based on the DSM-IV.^32,89^ Recent changes of diagnostic criteria in the DSM-5 seem to have minimal impact on the prevalence.^105^ Nonetheless, there should be a defined method to correct for different versions of the DSM.

This systematic review solely focused on the prevalence of PTSD as one of the most common psychiatric sequelae of TBI. However, Bryant and colleagues (2010) reported that only 8.9% of PTSD cases occurred without any comorbid disorders, and Alway^35^ confirmed this finding and showed that 93.3% of the participants with PTSD also experienced another psychiatric disorder. Other psychiatric comorbidities need to be taken into account when researching PTSD after TBI and exploring differences between prevalence rates. Another limitation concerns the impact of psychological treatment on the prevalence rates of PTSD in the included longitudinal studies. Half of the studies collected information on whether patients received psychological treatment for PTSD without specifying the type of treatment. None of them reported on the impact of a specific treatment on the prevalence rate of PTSD. For longitudinal studies in general, the timing of the outcome measures is important but subjects rarely present to assessment at precisely the allocated time-point. King and King suggest using individual time lags instead to avoid wide time-point intervals.^106^

In this review we chose to report prevalence rather than incidence. However, the use of prevalence versus incidence in this context may be debated. In some studies, researchers reported the incidence rate of PTSD but one cannot discard with certainty that another traumatic event caused the reported PTSD.^81^ This is especially relevant in military settings in which repeated trauma is common. Finally, this review did not consider biological factors, for example, the type of brain injury, and the influence of socioeconomic and cultural factors, which are other determining and influencing factors besides severity, instrument, and timing.^107^

### Moving ahead

This systematic review highlights substantial variability in reported findings between studies on PTSD and TBI in civilian settings. The relatively high number of studies graded as moderate to high risk of bias is of concern. The variability in methods may be in part due to quality aspects, but more likely reflects the heterogeneity not only of the population, but also of study designs, follow-up intervals, statistics, instruments, and other methodological factors.^108,109^ The large variability challenges the concept of a direct causal relation between TBI and PTSD—at least in civilian settings—and prevents the drawing of any strong conclusions. It is clear that much—and better—research is needed to progress the field. In moving the field forward, two important issues stand out: the need for higher quality studies and the need for better standardization. Recurrent issues have concerned the internal and external validity of studies. Future studies should report if they assessed sampling bias and addressed bias, as well as response rate, to assure internal validity. To cover external validity, studies require a clear definition of PTSD and need to report the measures used and their psychometric properties.

The NINDS (National Institute of Neurological Disorders and Stroke) Common Data Elements (CDE) strongly advocate improved standardization of data collection and reporting across all domains of research in TBI.^110^ Specific for post-traumatic stress symptoms, the NINDS recommends using the PCL and the CAPS as measurements for PTSD. We found in the reviewed studies that the PCL and the IES were the most commonly used screening instruments. The PCL was scored using the Symptoms Cluster Method (SCM) or by using a cutoff score between 36 and 44 for the TBI population.^111^ This is a lower cutoff score than that used in civilian primary care or general population samples (30–35) and higher than that used in mental health clinics (45–50).^10^ We suggest that a validated cutoff score needs to be determined for the recent version of the PCL-5 specific for the TBI population. Researchers should take the overlapping symptoms of PTSD and TBI into account when determining an accurate cutoff score. Symptoms of irritability, aggression, or negative thoughts might be an expression of PTSD or primarily be caused by the brain injury. The IES questionnaire can be an alternative screening test and is similar to the PCL. However, the IES only measures symptoms of two of the three clusters of PTSD.^86^ Studies used a cutoff score of 26 or 35 for preliminary diagnosis.^56,66,70,76,80^

The CAPS and the SCID were the most commonly used (semi-) structured interviews. Although the CAPS is considered the gold standard in measuring and diagnosing PTSD, and can better address any issue of overlapping symptoms,^78^ we found no clear difference in reported prevalence rates for PTSD between studies that used a screening tool or interview assessment of PTSD. It would appear that interview-based assessments of PTSD are not superior to screening instruments for PTSD in identifying the disorder. Besides standardization of assessment instruments, it is important to document the timing of assessment, the way of scoring, and interpretation of symptoms. For accurate diagnosis, professionals need to be trained and use standardized protocols. Adhering to these principles will hopefully increase the quality of future studies.

## Conclusion

This systematic review underlines the importance of screening for PTSD in patients with a TBI. The prevalence of PTSD after TBI in civilian populations is substantially lower than in combat settings, but remains high across studies. Health care professionals should be aware of the high risk of PTSD after TBI and should be able to recognize PTSD symptoms and distinguish these from typical symptoms of TBI. Use of a simple screening instrument can be considered as a first approach to identify those at risk for PTSD and facilitate prompt intervention. We found no association between the severity of TBI and occurrence of PTSD, and hence, recommend screening in patients with TBI of all severities. Neither did we find any clear effect of time of assessment on reported prevalence rates. The implication is that screening for PTSD remains relevant, even months to years after a TBI.

The association between PTSD symptoms and TBI is complex: the lack of a clear association between TBI severity and prevalence of PTSD raises the question of to what extent the brain injury itself plays a role in the development of PTSD and how the brain injury might affect the course of PTSD. How do consequences of TBI, such as cognitive deficiencies or problematic social re-integration interfere with PTSD and its treatment? Future research needs to focus on the event itself; pre-trauma- and patient-related factors, for example, personality factors; and post-trauma setting, for example, social support, taking into account the specific symptoms of TBI and its consequences.

Importantly, this review points to the need for high-quality studies and better standardization in research on PTSD following TBI, and specifically the recommendations of the NINDS CDEs should be followed. We suggest that studies should also include the population with chronic PTSD, in which social re-integration may be more challenging. Future investigation is necessary to develop and validate prognostic models to identify patients at high risk for PTSD following TBI. The sooner PTSD is diagnosed, the better it can be treated.^111^

## Supplementary Material

Supplemental data

Supplemental data

Supplemental data

Supplemental data

Supplemental data

Supplemental data

Supplemental data

Supplemental data
