# Supplementary material for: Post-Traumatic Stress Disorder after Civilian Traumatic Brain Injury: A Systematic Review and Meta-Analysis of Prevalence Rates
Source: J Neurotrauma. 2019 Nov 11;36(23):3220–32. doi: 10.1089/neu.2018.5759 (PMC6857464; doi:10.1089/neu.2018.5759)
Supplement: Supplemental data [file Supp_Appendix.docx]

Supplementary Appendix S1. Literature Search

Embase.com

('posttraumatic stress disorder'/exp OR (((posttrauma* OR trauma*) NEAR/3 (stress* OR psychic* OR neurosis OR psychosis OR psychoses OR psychosyndrome*)) OR ((posttraumat* OR traumat*) NEXT/1 (syndrome* OR symptom*)) OR ptsd OR ptss OR (stress* NEAR/3 (syndrom* OR symptom* OR scale* OR score*))):ab,ti) AND ('nervous system injury'/de OR 'brain injury'/de OR 'traumatic brain injury'/exp OR 'head injury'/de OR 'skull injury'/de OR (((brain OR head OR skull OR cerebral OR craniocerebral OR intracrani* OR cranial OR cns) NEAR/6 (injur* OR trauma* OR posttrauma* OR fracture* OR damage* OR defect* OR lesion* OR blast OR blunt)) OR tbi OR mtbi):ab,ti) NOT ([animals]/lim NOT [humans]/lim) NOT ('military phenomena'/exp/mj OR 'veteran'/de/mj OR 'soldier'/de/mj OR ((veteran* OR combat OR soldier* OR army OR war OR wars OR military OR militaries):ti ) NOT (civilian* OR (general NEAR/3 population*) OR nonmilitar* OR (non NEXT/1 militar*)):ab,ti)

Medline ovid

("Stress Disorders, Post-Traumatic"/ OR (((posttrauma* OR trauma*) ADJ3 (stress* OR psychic* OR neurosis OR psychosis OR psychoses OR psychosyndrome*)) OR ((posttraumat* OR traumat*) ADJ (syndrome* OR symptom*)) OR ptsd OR ptss OR (stress* ADJ3 (syndrom* OR symptom* OR scale* OR score*))).ab,ti.) AND ("Trauma, Nervous System"/ OR exp "Craniocerebral Trauma"/ OR (((brain OR head OR skull OR cerebral OR craniocerebral OR intracrani* OR cranial OR cns) ADJ6 (injur* OR trauma* OR posttrauma* OR fracture* OR damage* OR defect* OR lesion* OR blast OR blunt)) OR tbi OR mtbi).ab,ti.) NOT (exp animals/ NOT humans/) NOT (* "Military Personnel"/ OR * "Veterans"/ OR * "Warfare and Armed Conflicts"/ OR * "Combat Disorders"/ OR ((veteran* OR combat OR soldier* OR army OR war OR wars OR military OR militaries).ti. ) NOT (civilian* OR (general ADJ3 population*) OR nonmilitar* OR (non ADJ militar*)).ab,ti.)

PsycINFOovid

("Posttraumatic Stress Disorder"/ OR (((posttrauma* OR trauma*) ADJ3 (stress* OR psychic* OR neurosis OR psychosis OR psychoses OR psychosyndrome*)) OR ((posttraumat* OR traumat*) ADJ (syndrome* OR symptom*)) OR ptsd OR ptss OR (stress* ADJ3 (syndrom* OR symptom* OR scale* OR score*))).ab,ti.) AND ("Traumatic Brain Injury"/ OR exp "Brain Damage"/ OR (((brain OR head OR skull OR cerebral OR craniocerebral OR intracrani* OR cranial OR cns) ADJ6 (injur* OR trauma* OR posttrauma* OR fracture* OR damage* OR defect* OR lesion* OR blast OR blunt)) OR tbi OR mtbi).ab,ti.) NOT (exp animals/ NOT humans/) NOT (* "Military Personnel"/ OR * "Military Veterans"/ OR * "War"/ OR * "Combat Experience"/ OR ((veteran* OR combat OR soldier* OR army OR war OR wars OR military OR militaries).ti. ) NOT (civilian* OR (general ADJ3 population*) OR nonmilitar* OR (non ADJ militar*)).ab,ti.)

Cinahlebsco

(MH "Stress Disorders, Post-Traumatic" OR (((posttrauma* OR trauma*) N2 (stress* OR psychic* OR neurosis OR psychosis OR psychoses OR psychosyndrome*)) OR ((posttraumat* OR traumat*) N1 (syndrome* OR symptom*)) OR ptsd OR ptss OR (stress* N2 (syndrom* OR symptom* OR scale* OR score*)))) AND (MH "Head Injuries" OR MH "Brain Injuries+" OR (((brain OR head OR skull OR cerebral OR craniocerebral OR intracrani* OR cranial OR cns) N5 (injur* OR trauma* OR posttrauma* OR fracture* OR damage* OR defect* OR lesion* OR blast OR blunt)) OR tbi OR mtbi)) NOT (MH animals+ NOT MH humans) NOT (MM "Military Personnel+" OR MM "Veterans+" OR MM "War+" OR (TI (veteran* OR combat OR soldier* OR army OR war OR wars OR military OR militaries) ) NOT (civilian* OR (general N2 population*) OR nonmilitar* OR (non N1 militar*)))

Cochrane

((((posttrauma* OR trauma*) NEAR/3 (stress* OR psychic* OR neurosis OR psychosis OR psychoses OR psychosyndrome*)) OR ((posttraumat* OR traumat*) NEXT/1 (syndrome* OR symptom*)) OR ptsd OR ptss OR (stress* NEAR/3 (syndrom* OR symptom* OR scale* OR score*))):ab,ti) AND ((((brain OR head OR skull OR cerebral OR craniocerebral OR intracrani* OR cranial OR cns) NEAR/6 (injur* OR trauma* OR posttrauma* OR fracture* OR damage* OR defect* OR lesion* OR blast OR blunt)) OR tbi OR mtbi):ab,ti) NOT (((veteran* OR combat OR soldier* OR army OR war OR wars OR military OR militaries):ti ) NOT (civilian* OR (general NEAR/3 population*) OR nonmilitar* OR (non NEXT/1 militar*)):ab,ti)

Web-of-science

TS=(((((posttrauma* OR trauma*) NEAR/2 (stress* OR psychic* OR neurosis OR psychosis OR psychoses OR psychosyndrome*)) OR ptsd OR ptss OR (stress* NEAR/2 (syndrom* OR symptom* OR scale* OR score*)))) AND ((((brain OR head OR skull OR cerebral OR craniocerebral OR intracrani* OR cranial OR cns) NEAR/5 (injur* OR trauma* OR posttrauma* OR fracture* OR damage* OR defect* OR lesion* OR blast OR blunt)) OR tbi OR mtbi)) NOT ((animal* OR rat OR rats OR mouse OR mice OR murine) NOT (human* OR patient*))) NOT ((TI=(veteran* OR combat OR soldier* OR army OR war OR wars OR military OR militaries) ) NOT TS=(civilian* OR (general NEAR/2 population*) OR nonmilitar* OR (non NEAR/1 militar*)))

Pubmed publisher

("Stress Disorders, Post-Traumatic"[mh] OR (posttraumatic syndrome*[tiab] OR post-traumatic syndrome*[tiab] OR posttraumatic symptom*[tiab] OR post-traumatic symptom*[tiab] OR posttraumatic stress*[tiab] OR post-traumatic stress*[tiab] OR posttraumatic psychic*[tiab] OR post-traumatic psychic*[tiab] OR posttraumatic neuros*[tiab] OR post-traumatic neuros*[tiab] OR posttraumatic psychosis*[tiab] OR post-traumatic psychosis*[tiab] OR posttraumatic psychoses*[tiab] OR post-traumatic psychoses*[tiab] OR ptsd OR ptss )) AND ("Trauma, Nervous System"[mh] OR "Craniocerebral Trauma"[mh] OR (brain trauma* OR traumatic brain injur* OR head trauma* OR cerebral trauma* OR craniocerebral trauma* OR intracranial trauma*[tiab] OR cranial trauma* OR tbi OR mtbi)) NOT (animals[mh] NOT humans[mh]) NOT ("Military Personnel"[mj] OR "Veterans"[mj] OR "Warfare and Armed Conflicts"[mj] OR "Combat Disorders"[mj] OR ((veteran*[ti] OR combat[ti] OR soldier*[ti] OR army[ti] OR war[ti] OR wars[ti] OR military[ti] OR militaries[ti]) ) NOT (civilian*[tiab] OR (general population*[tiab]) OR nonmilitar*[tiab] OR (non militar*[tiab]))) AND publisher[sb]

Google scholar

ptsd|ptss "brain|head|skull|cerebral|craniocerebral|intracranial|cranialinjury|trauma|fracture|damage|blast|blunt"|tbi|mtbi -veteran -veterans -combat -soldier -war -military

Opengrey.eu

Handsearch (use criteria google scholar)

Behavioral and Brain sciences – Cambridge university press

Handsearch (use criteria google scholar)

BioMed Central and National Institute of Mental Health

Handsearch (use criteria google scholar)

Reference lists of included papers

Handsearch after the initial selection of papers
