# Supplementary figures and images for: Post-Traumatic Stress Disorder after Civilian Traumatic Brain Injury: A Systematic Review and Meta-Analysis of Prevalence Rates
Source: J Neurotrauma. 2019 Nov 11;36(23):3220–32. doi: 10.1089/neu.2018.5759 (PMC6857464; doi:10.1089/neu.2018.5759)

Supplementary Fig. S1. Study Selection


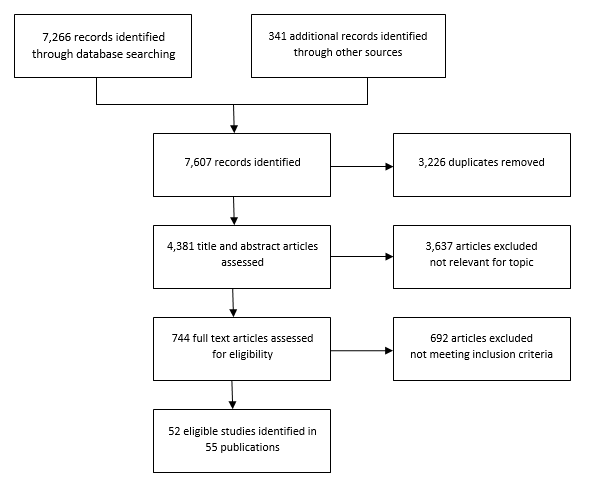

Supplement: Supplemental data [file Supp_Fig1.docx]

Supplementary Fig. S2.1. Meta-analysis of PTSD in Mild TBI


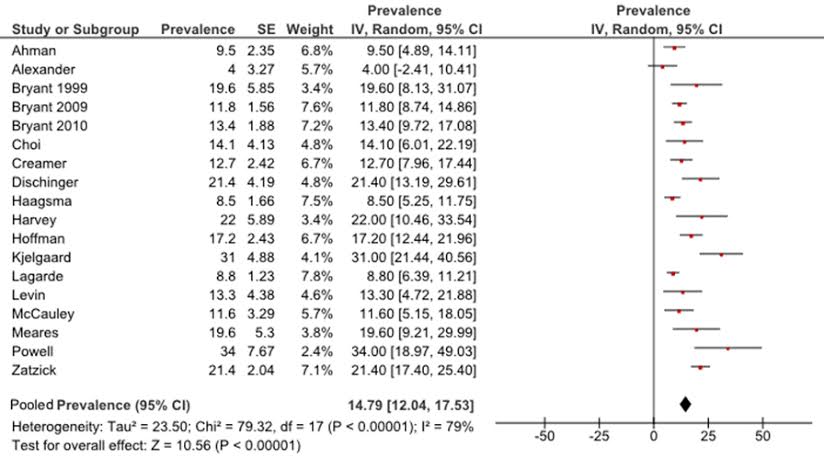

Supplement: Supplemental data [file Supp_Fig2-1.docx]

Supplementary Fig. S2.2. Meta-analysis of PTSD in Moderate and Severe TBI


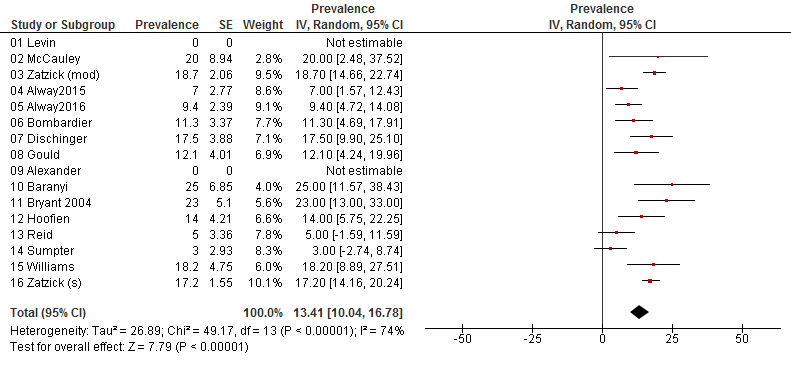

Supplement: Supplemental data [file Supp_Fig2-2.docx]

Supplementary Fig. S2.3. Meta-analysis of PTSD in TBI (mixed severity) in Low Risk of Bias Studies


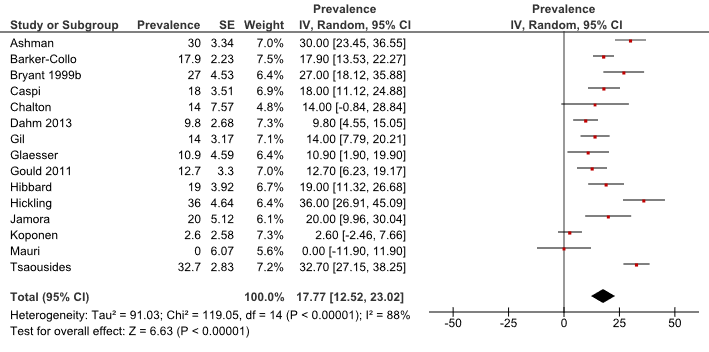

Supplement: Supplemental data [file Supp_Fig2-3.docx]

Supplementary Fig. S2.4. Meta-analysis of PTSD in TBI (mixed severity)


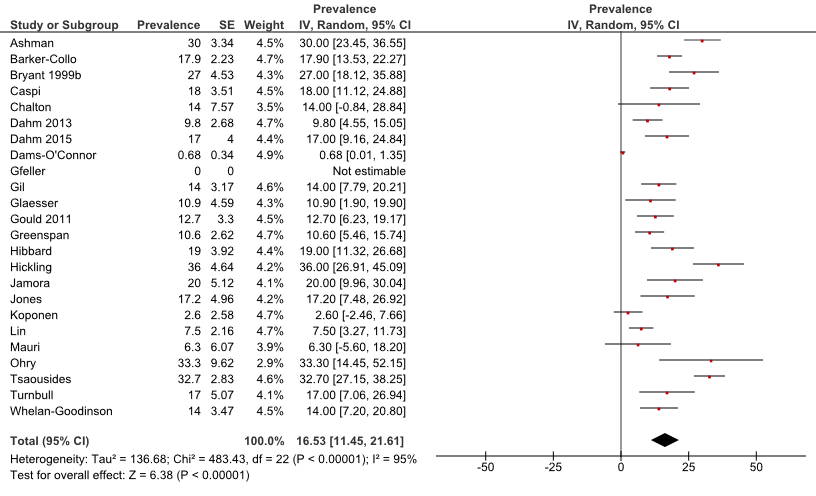

Supplement: Supplemental data [file Supp_Fig2-4.docx]
