# Supplementary material for: Post-Traumatic Stress Disorder after Civilian Traumatic Brain Injury: A Systematic Review and Meta-Analysis of Prevalence Rates
Source: J Neurotrauma. 2019 Nov 11;36(23):3220–32. doi: 10.1089/neu.2018.5759 (PMC6857464; doi:10.1089/neu.2018.5759)
Supplement: Supplemental data [file Supp_Table1.docx]

Supplementary Table S1. Study Characteristics

| **Authors, year, country, design** | **Study population** | **Study sample Inclusion/exclusion,  age, N (Response)** | **Instrument** | **Assessment method  Timing** | **Outcome % PTSD** |
| --- | --- | --- | --- | --- | --- |
| Ahman, 2013, Sweden ^A,66^ | mTBI | arrived at ED <24h,  18-64y, n=163 | IES | Questionnaire 3y, 11y | 9.5% of men, 13.2% of women |
| Alexander, 1992, USA ^A,50^ | TBI only closed head injury | Closed head injury, injury within 5y, CT or MRI, no patients that received rehab <6m, n=36 | Psychiatric interview | Interview | 3% of TBI (4% mTBI, 0% STBI) |
| Alway, 2015, Australia ^A,35^ | modTBI, sTBI | PTA>24h, no history of TBI or another neurological disorder, residing in Australia, sufficient cognitive and English proficiency  16-80y, n=85 | SCID-I | Interview  0m, 6m, 1y, 2y, 3y, 4y | 0m: 1.1%, 6m: 4.7%, 12m: 7%, 2y: 2.4%, 3y: 2.4%, 4y: 17.65% |
| Alway, 2016, Australia ^A,36^ | modTBI, sTBI | PTA>24h, no history of TBI or another neurological disorder, residing in Australia, sufficient cognitive and English proficiency  16-76y, n=203 | SCID-I | Interview  0m, 3m, 6m, 12m, 2y, 3y, 4y, 5y | 0m: 0.4%, 3m: 1.9%, 6m: 4.3%, 12m: 9.4%, 2y: 8.89%, 3y: 8.69%, 4y: 5.60%, 5y: 5.0% |
| Ashman, 2004, USA ^D,51^ | TBI | 3m to 4y postinjury, resident of US, capable of giving informed consent, no other brain injury, preexisting neurocognitive or psychotic disorder  18-87y, n=188 | SCID-I | Interview  3m to 3y, 12m, 24m | initial 3m to 3y: 30%, 12m: 18%, 24m: 21% |
| Baranyi,2010, Austria ^D,67^ | sTBI + severelyinjurednoTBI | ISS > 11 15-70y, n=52 (TBIn=40) | SCID-I, CAPS, IES | Interview/questionnaire 12m | 19.2%, noTB:I 5.8% |
| Barker-Collo, 2013, Australia ^A,37^ | TBI | Confusion, disorientation, LOC ,PTA, or other neurological abnormalities, primarily related to TBI, written consent, 16+y, n=296 | PDS | Questionnaire 12m | 17.9% |
| Bombardier, 2006, USA ^A,52^ | modTBI, sTBI | Radiological evidence for TBI or lowest GCS < 13, residing in King, Pierce, Kitsap or Snohomish counties, English, no homeless or incarcerated patients, no history of schizophrenia, or participation in investigational drug study 18+y, n=81 | PCL-C | Questionnaire 1m, 2m, 3m, 4m, 5m or 6m | 11.3% (within first 6 months) |
| Bryant, 1999a, Australia ^A,38^ | mTBI + noTBI | LOC, PTA <24h, English, medically fit, no narcotic analgesia, ability to contact patient 16-65y, n=105 (TBIn=46) | CIDI | Interview  6m, 1y, 2y, 3y, 4y | 19.6%, noTBI: 25.4% |
| Bryant, 1999b, Australia ^A,39^ | TBI | English, sufficient cognitive ability for interview, consent, ability to contact patient 16-71y, n=96 | PTSD-I | Interview 6m | 27% |
| Bryant, 2004, Australia ^A,40^ | sTBI | English, sufficient cognitive ability for interview, consent, ability to contact patient, available medical record n=68 | PTSD-I | Interview 6m | 23% |
| Bryant, 2009, Australia ^A,41^ | mTBI + noTBI | Documented head injury, LOC <30min, PTA <24h, English, admission >24h, no modTBI or sTBI, no psychotic, suicidal, non-australian patients, or under police guard 16-70y, n=920 | CAPS-IV | Interview  3m | 11.8%, noTBI: 7.5% |
| Bryant, 2010, Australia, ^A,7^ | mTBI + noTBI | mTBI defined by ICD9, admission >24h, English, no psychotic, suicidal, non-australian patients, or under police guard 16-70y, n=932 (TBIn=377) | CAPS | Interview  3m, 12m | 12.7%, noTBI: 7.6%, 12m: 13.4%, noTBI 7.2% |
| Caspi, 2005, Israel^D,97^ | TBI | Hebrew, no history of brain damage or other chronic medical conditions, no history of major psychiatric illnesses, substance abuse, or cognitive deficits 18-50y, n=120 | SCID-I, CAPS | Interview  2.9y to 3.7y | 18% |
| Chalton, 2009, UK ^D,68^ | TBI | head injury within 3m, no psychological treatment  20-65y, n=21 | PDS, CAPS | Questionnaire 3m to 359m | PDS: 33%, CAPS: 14% |
| Choi, 2014, Korea ^D,81^ | mTBI | serial CT, sufficient communication 17-84y, n=71 | PCL-C | Questionnaire 2.2m to 8m | 14.1% (2.8% confirmed) |
| Creamer, 2005, Australia ^A,42^ | mTBI + noTBI | admission >24h, English, no self-harm, no IV drugs or psychiatric disorder 18-70y, n=307 (TBIn=189) | CAPS-IV | Interview  12m | 12.7%, noTBI: 7% |
| Dahm, 2013, Australia ^D,43^ | TBI | English, sufficient cognitive abilities, no other neurological disorders, consent  18-78y, n=123 | SCID-I | Interview  2m to 25y | 9.8% |
| Dahm, 2015, Australia ^A,44^ | TBI + TraumaticOrthopedicInjury | English, sufficient cognitive abilities, no sustained spinal cord injuiries, major burns, or traumatic amputations. For TBI group, no other neurological disorders. For TOI group, no sustained brain injury other than uncomplicated mild TBI,  16-77.4y (TOI 17.7-84.4y), n=184 (TBIn=88) | PCL-S | Questionnaire 5y to 10y | 17%, noTBI: 5% |
| Dams-O'Connor, 2013, USA ^A,53^ | TBI | ED <24h, CT consent, English, no pregnant patients, in custody, or in process of psychiatric evaluation, excluded when contra-indications to MRI mean 41.23y (TOI 43.97y), n=586 | PCL-C | Questionnaire 3m or 6m | 0.68% (0.4% with no previous TBI, 1.5% with previous TBI) |
| Dischinger, 2003, USA ^B,54^ | mTBI + modTBI or sTBI | motor vehicle crash  16-60+y, n=96 | 'questionsdiagnostic of PTSD' | Interview  6m, 12m | mTBI: 19.6%, mod/sTBI: 27.5%, 12m mildTBI: 21.4%, mod/sTBI: 17.5% |
| Gfeller, 2013, USA ^C,55^ | chronic TBI + military TBI + civilian TBI | English, no history of intellectual disability, no contradictory pre-existing conditions. For control, no history o psychiatric illness, neurological illness, substance use disorder or other cognitive or developmental disorders, 18+y, n= 60 (TBIn=40) | PCL-C | Questionnaire | cTBI: 0%, vTBI: 45%, noTBI: 0% |
| Gil, 2005, Israel^A,109^ | TBI | Hebrew, no psychiatric care, cognitive deficits, substance abuse or major untreated medical conditions 18-50y, n=120 | CAPS, PSS | Interview/questionnaire 6m | 14% |
| Glaesser, 2004, Germany ^D,69^ | TBI | inpatients from neurological rehabilitation unit, TBI within 5y, admitted over a period of 4m 19-58y, n=46 | SCID-I | Interview  1m to 82m | 10.9% |
| Gould, 2011, Australia ^A,45^ | TBI | complicated mTBI, mod or sTBI, no previous TBI or other neurological disorder, residence in Australia, English, sufficient cognitive ability  16-73y, n=102 | SCID-I | Interview  12m | 12.7% |
| Gould, 2014, Australia ^A,46^ | modTBI, sTBI | no previous TBI or other neurological disorder, no learning disorder or ADHD, residence in Australia, English, sufficient cognitive ability, 18-61y, n=66 | SCID-I | Interview  12m | 12.1% |
| Greenspan, 2006, USA ^A,56^ | TBI | sustained damage to brain tissue due to an external force, presented <24h, English or Spanish 16+y, n=198 | IES | Questionnaire 6m, 12m | 6m: 10.6%, 12m: 16.2% |
| Haagsma, 2015, the Netherlands ^A,70^ | mTBI | admitted to ED of RUNMC, consent, Dutch, possibility of follow-up, no alcohol or drug abuse or dementia 16+y, n=797 | IES | Questionnaire 6m, 12m | 6m: 8.7%, 12m: 8.5% |
| Harvey, 2000, Australia ^A,47^ | mTBI | motor vehicle accidents 17-58y, n=63 | CIDI | Interview  6m, 2y | 6m: 24%, 2y: 22% |
| Hibbard, 1998, USA^D,57^ | TBI | injury within 1y, traumatic origin, resident of NY, living in the community  18-59y, n=100 | SCID-I | Interview  mean 7.6y | 19% |
| Hickling, 1998, USA ^A,58^ | TBI only motor vehicle accidents | Motor vehicle accidents, injury within 2 days n=107 | CAPS | Interview  1m to 4m | 36% |
| Hoffman, 2012, USA ^B,59^ | mTBI | Injury within 48h, LOC <30min, impaired consciousness >24h, or PTA <24h, English, permanent home address and phone number, no abnormal findings on CT or admission to ICU, no other serious injuries, neurologic disease or end-stage terminal disease, no major psychiatric illness, no injuries received during sexual assault, no hospitalization > 2 days in the past year, no alcohol abuse or dependence on drugs, no prisoners or in custody 16+y, n=239 | PCL-C | Questionnaire 6m | 17.2% |
| Hoofien, 2001, Israel^D,82^ | sTBI | 17-50y, n=68 | PTSD-I | Interview mean 14.1y | 14% |
| Jamora, 2012, USA ^D,60^ | TBI | English, questionable effort on TMM, no neurological or psychiatric history, no premorbid TBI, no atypical responses on RNBI validity scales, no severe sensory limitations 16+y, n=61 | RNBI | Questionnaire mean 25.47m | 20% |
| Jones, 2005, UK ^A,71^ | TBI + noTBI | English, medically well enough within 14 days, resident within 30 km of Oxford, PTA <24h, not under influence of alcohol or drugs, no treatment for psychological disorder, RTA must not have involved a fatality 18-65y, n=119 (TBIn=58) | PSS | Interview 3m | 17.2%, noTBI: 18% |
| Kjeldgaard, 2014, Denmark ^D,72^ | Chronic PostTraumatic Headache after mild injury + primary headache | Diagnosis with CPTH attributed to mild injury, interested in possible psychological treatment of their headache, no other neurological or psychiatric disorders, no pregnancy, substance overuse, or pre-existing primary headache, no CPTH after whiplash 18-65y, n=135 (TBIn=90) | HTQ | Questionnaire | 31% |
| Koponen, 2011, Finland ^A,73^ | TBI | Acute brain trauma, LOC >1 min, PTA >30 min, neurological symptoms or neuroradiological findings indicating TBI, no other central nervous system diseases 16-67y, n=38 | SCAN | Interview  12m | 2.6% |
| Lagarde, 2014, France ^A,74^ | mTBI + noTBI | Only patients with severity criterion, presented to ED <24h after injury, living in France, French, no patients with severe injury or other neurological disease, history of motor neuron disease or a cerebral vascular accident, no homeless patients or unable to answer the questionnaire for medical reasons 15+y, n=1631 (TBIn=534) | standardized questionnaire | Questionnaire 3m | 8.8%, noTBI: 2.2% |
| Levin, 2001, USA ^A,61^ | mTBI + modTBI + general trauma | mTBI: closed head injury, LOC <20 min, lowest GCS of 13-15, no extracranial injury, CT <24h, no surgery. ModTBI: lowest GCS was 9-12, residence within Harris County, no penetrating missile injury of the brain, history of diagnosed schizophrenia, mental deficiency, hospitalization for previous TBI, history of treatment for substance abuse, high blood alcohol level 16+y, n=111 (TBIn=69) | SCID-I, PCL | Interview  3m | mTBI: 13.3%, modTBI: 0%, TBI: 11.6%, noTBI: 11.5% |
| Lin, 2014, Taiwan ^A,83^ | TBI + fracture, open wound of upper limb, crushing injury, burns | n=1025 (TBIn=149) | MINI, PTSC | Interview/questionnaire 12m | 7.5%, crushing injuries: 0%, open wound of upper limbs: 1.6%, fracture: 2.6%, burns: 3.7% |
| Mauri, 2014, Italy ^C,75^ | TBI + matched control group | lesion on CT, LOC >1 min, PTA >30 min, neuroradiological evidence, no unstable neurological or cardiorespiratory conditions, past psychiatric diagnosis or substance abuse 18-65y, n=22 (TBIn=16) | SCID-I | Interview  1m, 3m to 6m | 1m: 6.25%, 3m: 6.25%, 6m: 0% |
| McCauley, 2001, USA ^A,62^ | mTBI, modTBI + general trauma | non-penetrating head injury, CT <24h of injury, English or Spanish, no previous hospitalization for head trauma, no preinjury major neuropsychiatric disorder, preinjury substance dependence, pre-existing CNS disturbance, associated spinal cord injury, undocumented resident status. For TBI group:no high blood alcohol level or surgery with general anesthesia 16+y, n=200 (TBIn=115) | SCID-I, PCL | Interview/questionnaire 3m | mTBI: 11.6%, modTBI: 20%, TBI: 13%, noTBI: 14.1% |
| Meares, 2011, Australia ^A,48^ | mTBI + trauma controls | admission <24h, assessment <14 days, follow up <5m, effort on testing based on WMT, IQ >70, English, no modTBI or sTBI, or mTBI and sustained an intracranial lesion, no self harm, psychotic, pre-existing cognitive impairment, medically fit, no interstate or oversease visitor, no pregnant patients or subject of forensic investigation,  18-65y, n=120 (TBIn=62) | CAPS | Interview  3m | 19.6%, noTBI: 10.3% |
| Ohry, 1996, USA ^A,63^ | TBI | in rehabilitation 16-67y, n=24 | PTSD-I, IES | Questionnaire 6m to 8m | 33.3% |
| Powell, 1996, UK ^A,76^ | minor headinjury | minor head injury, no subsequent deterioration or neurosurgical intervention, LOC <20min, PTA <24h, no neuropsychiatric problems 18-79y, n=35 | IES | Questionnaire 3m | 34% |
| Reid, 2011, UK ^D,77^ | sTBI | IQ >79, not color blind, severe head injury over 3 months post injury, living independently 17+y, n=42 | CAPS | Interview  minimum 3m | 5% |
| Sumpter, 2005, UK ^D,78^ | sTBI | >3 months postinjury, MMSE >27, no severe dysphasia or dyslexia, or current treatment for psychosis 20-60y, n=34 | CAPS, IES, PDS | Interview/questionnaire minimum 3m | 3% |
| Tsaousides, 2011, USA ^A,64^ | TBI | community dwelling patients with history of TBI, >3 months post injury, consent, able to answer questions independently, no nontraumatic brain injury or preexisting neurocognitive or psychotic disorder 18+y, n=275 | SCID-I | Interview  minimum 3m | 32.7% (29.9% no suicidal ideation, 39.1% suicidal ideation) |
| Turnbull, 2001, Scotland ^D,79^ | TBI | LOC, PTA, head injury in previous 6 months, traumatic event, evidence of TBI, no chronic alcohol abuse 16-65y, n=55 | IES-R, CAPS-DX | Interview/questionnaire 0m to 6m | 17% using stringent criteria and 27% using lenient criteria |
| Whelan-Goodinson, 2009, Australia ^B,49^ | TBI | English, no history of previous TBI or serious neurological or psychiatric disorders  19-74y, n=100 | SCID-I | Interview  6m to 5.5y | 14% |
| Williams, 2002, UK ^D,80^ | sTBI | 17-70y, n=66 | IES | Questionnaire 1y to 26y | 18.2% (consisting of subclinical 54.5%, mild 27.3%, moderate 12,1%, severe 6,1%) |
| Zatzick, 2010, USA ^A,65^ | TBI + noTBI | English or Spanish, not incarcerated, no treatment delays >24h, hip francture or majur burn  18-65y, n=2993 (TBIn=1356) | PCL-C | Questionnaire 12m | mTBI: 21.4%, modTBI: 18.7%, sTBI: 17.2%, noTBI: 21.1% |
| A Prospective cohort study, B Retrospective cohort study, C Case control study, D Cross sectional study | | | | | |
