# Supplementary material for: Post-Traumatic Stress Disorder after Civilian Traumatic Brain Injury: A Systematic Review and Meta-Analysis of Prevalence Rates
Source: J Neurotrauma. 2019 Nov 11;36(23):3220–32. doi: 10.1089/neu.2018.5759 (PMC6857464; doi:10.1089/neu.2018.5759)
Supplement: Supplemental data [file Supp_Table2.docx]

| Supplementary Table S2. Risk of Bias | | | | | | | | | | | | |
| --- | --- | --- | --- | --- | --- | --- | --- | --- | --- | --- | --- | --- |
| Study | 1 Subjectsampling | 2 Assessment of samplingbias | 3 Estimatebias | 4 Exclusion rate from the analysis | 5 Address Bias | 1 Source of measure | 2 Definition of PTSD | 3 Validity | 4 Reliability | 5.1 Reporting of prevalence | 5.2 Precision of estimate | Ri  sk of bias |
| Ahman, 2013^66^ | + | + | + | + | + | + | - - | + | PR | + | PR | Moderate |
| Alexander, 1992^50^ | + | + | PR | + | + | + | + | - - | PR | + | PR | Moderate |
| Alway, 2015^35^ | + | + | + | - - | + | + | + | + | PR | + | PR | Moderate |
| Alway, 2016^36^ | + | + | + | + | + | + | + | + | PR | + | PR | No |
| Ashman, 2004^51^ | + | + | + | + | + | + | + | + | + | + | PR | No |
| Baranyi, 2010^67^ | + | + | + | + | + | + | + | + | PR | + | PR | No |
| Barker-Collo, 2013^37^ | + | + | + | + | + | + | + | + | + | + | PR | No |
| Bombardier, 2006^52^ | + | + | + | + | + | + | + | + | + | + | PR | No |
| Bryant, 1999a^38^ | + | + | + | + | + | + | + | + | PR | + | PR | No |
| Bryant, 1999b^39^ | + | + | + | + | - | + | + | + | PR | + | PR | No |
| Bryant, 2004^40^ | + | + | + | + | - | + | + | + | + | + | PR | No |
| Bryant, 2009^41^ | + | + | + | + | + | + | + | + | PR | + | + | No |
| Bryant, 2010^7^ | + | + | + | + | + | + | + | + | + | + | + | No |
| Caspi, 2005^97^ | + | PR | + | + | PR | + | + | + | PR | + | PR | No |
| Chalton, 2009^68^ | + | PR | PR | + | PR | + | + | + | PR | + | PR | No |
| Choi, 2014^81^ | + | PR | PR | + | PR | + | + | - - | PR | + | PR | Moderate |
| Creamer, 2005^42^ | + | PR | + | + | PR | + | + | + | + | + | PR | No |
| Dahm, 2013^43^ | + | + | + | + | + | + | + | + | PR | + | PR | No |
| Dahm, 2015^44^ | + | + | - - | + | + | + | + | + | PR | + | PR | Moderate |
| Dams-O'Connor, 2013^53^ | + | + | PR | + | PR | + | - - | + | PR | + | PR | Moderate |
| Dischinger, 2003^54^ | - - | + | - - | + | PR | + | PR | - - | PR | + | PR | High |
| Gfeller, 2013^55^ | + | + | PR | - - | + | + | - - | + | PR | + | PR | High |
| Gil, 2005^109^ | + | PR | + | + | PR | + | + | + | PR | + | PR | No |
| Glaesser, 2004^69^ | + | + | + | + | PR | + | + | + | PR | + | PR | No |
| Gould, 2011^45^ | + | + | PR | PR | + | + | + | + | + | + | PR | No |
| Gould, 2014^46^ | + | + | PR | PR | + | + | + | + | PR | + | PR | No |
| Greenspan, 2006^56^ | - | + | PR | PR | + | + | - - | + | PR | + | PR | Moderate |
| Haagsma, 2015^70^ | + | + | + | + | PR | + | - - | + | PR | + | PR | Moderate |
| Harvey, 2000^47^ | - | PR | PR | + | PR | + | + | + | PR | + | PR | No |
| Hibbard, 1998^57^ | + | + | PR | + | PR | + | + | + | PR | + | PR | No |
| Hickling, 1998^58^ | - | PR | PR | + | PR | + | + | + | PR | + | PR | No |
| Hoffman, 2012^59^ | + | + | + | + | + | + | + | + | PR | + | PR | No |
| Hoofien, 2001^82^ | + | PR | PR | + | PR | + | + | + | PR | + | PR | No |
| Jamora, 2012^60^ | + | + | + | + | + | + | + | + | + | + | PR | No |
| Jones, 2005^71^ | + | + | - - | PR | + | + | - - | + | + | + | PR | High |
| Kjeldgaard, 2014^72^ | + | + | + | + | + | + | - - | + | PR | + | PR | Moderate |
| Koponen, 2011^73^ | + | PR | + | + | PR | + | + | + | PR | + | + | No |
| Lagarde, 2014^74^ | + | + | + | + | PR | + | + | - - | PR | + | PR | Moderate |
| Levin, 2001^61^ | + | + | PR | + | + | + | + | + | PR | + | PR | No |
| Lin, 2014^83^ | - - | PR | - - | + | + | + | + | + | PR | + | + | High |
| Mauri, 2014^75^ | + | + | + | + | + | + | + | + | PR | + | PR | No |
| McCauley, 2001^62^ | + | + | PR | + | + | + | + | + | PR | + | PR | No |
| Meares, 2011^48^ | + | + | + | - - | + | + | + | + | + | + | PR | Moderate |
| Ohry, 1996^63^ | - - | + | PR | + | + | + | + | + | PR | + | PR | Moderate |
| Powell, 1996^76^ | - | + | PR | + | + | + | - - | + | PR | PR | PR | Moderate |
| Reid, 2011^77^ | + | PR | PR | + | PR | + | + | + | PR | + | PR | No |
| Sumpter, 2005^78^ | + | PR | PR | + | PR | + | + | + | PR | + | PR | No |
| Tsaousides, 2011^64^ | + | PR | PR | + | PR | + | + | + | PR | + | PR | No |
| Turnbull, 2001^79^ | + | + | - - | + | + | + | - - | + | PR | + | PR | High |
| Whelan-Goodinson, 2009^49^ | + | + | - - | + | + | + | + | + | + | + | PR | Moderate |
| Williams, 2002^80^ | + | PR | PR | + | PR | + | - - | + | PR | + | PR | Moderate |
| Zatzick, 2010^65^ | + | + | + | + | + | + | - - | + | + | + | + | Moderate |
